# Characterization of the relationship between bicuspid aortic valve morphology and hemodynamics

**DOI:** 10.1186/1532-429X-17-S1-O96

**Published:** 2015-02-03

**Authors:** Vrishank Raghav, Daniel Mangiameli, Elizabeth Coco, Alex J Barker, Michael Markl, Ajit P Yoganathan

**Affiliations:** 1 Georgia Institute of Technology, Atlanta, GA, USA; 2 Northwestern University, Chicago, IL, USA

## Background

Bicuspid aortic valve (BAV) is the most common congenital heart defect affecting 1-2% of the US population. BAV has been associated with progressive secondary pathologies, such as aortic valve calcification, stenosis, regurgitation, aortic root dilatation, and/or aortic aneurysm. However, not all patients exhibit symptoms or develop aortopathy and the exact link between BAV morphology and disease progression is not fully understood. Magnetic resonance imaging (MRI) has been previously used to characterize BAV hemodynamics. However, studies have been hampered by the need for lengthy manual data analysis thereby limiting reproducibility and/or integration into a clinically feasible workflow.

## Methods

**Figure 1:**
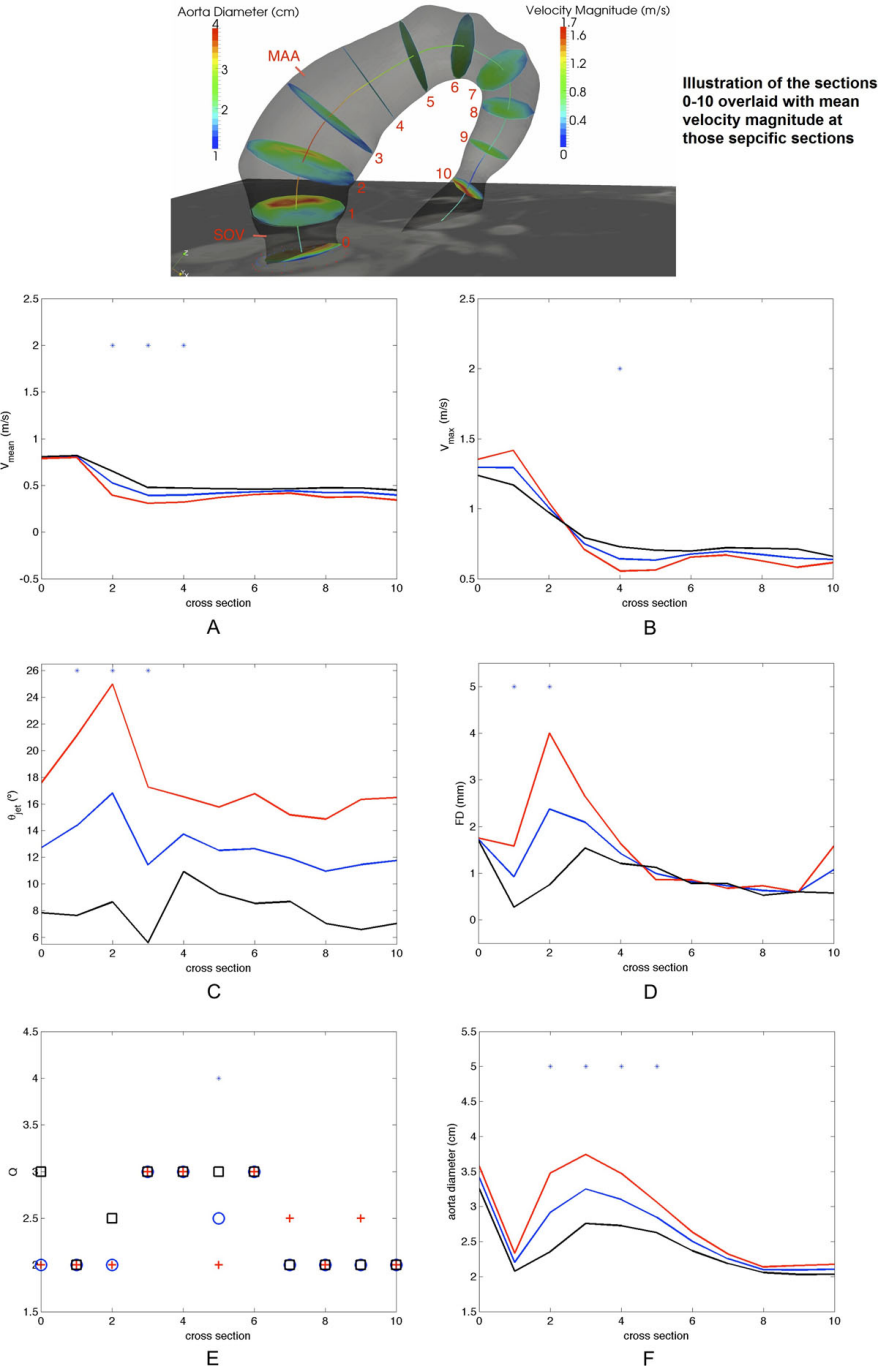
Jet quadrant is express in terms of its median. Red continuous line and "plus" marker: BAV subjects; black continuous line and "square" marker: TAV subjects; blue continuous line and "circular" marker: all subjects. The marker ‘*' indicates the section with a statistically significant difference between BAV and TAV groups (p<0.05). 1a: mean velocity; 1b: maximum velocity; 1c: jet angle; 1d: forward flow eccentricity; 1e: jet quadrant; 1f: area of aorta cross-sections.

This work develops and applies a novel semi-automated technique to characterize geometry and hemodynamics of the aorta and the aortic valve based on 2D bSSFP cine and 4D flow MRI data. The protocol was applied to a cohort of 30 BAV and 30 control subjects. The table summarizes the preliminary analysis (performed on a 12 by 12 subset of the original 30 by 30 cohort) of the demographics of the subject cohort. The analysis was performed at several cross-sectional locations across the ascending aorta (see figure 1). Statistical differences between the groups were identified and linear regression models were developed to elucidate the potential of this protocol to investigate the risk of disease progression.

## Results

Geometric metrics (summarized in the table) were found to be significantly larger in the BAV subjects than in the control group (preliminary results are summarized in table 1). The figure illustrates a summary of the preliminary analysis for mean velocity, valve outflow jet angle and flow displacement across the different sections of the ascending aorta. Key findings with respect to hemodynamics include the correlation in the proximal ascending aorta between aorta diameter and mean velocity (negative), jet angle (positive) and flow displacement (positive), in the combined cohort.

**Table 1:** Demographic information of the subjects cohort (RL: right-left fusion; RN: right-non coronary fusion; LN: left-non coronary fusion; SOV: diameter at the sinuses of Valsalva; MAA: diameter at the mid-ascending aorta)

	BAV	TAV	p-value
N	12	12	-

Age (years)	48 ± 13	45 ± 12	0.55

Female	3	5	-

Fusion	RL: 9; LN: 1; RN: 2	-	-

SOV (cm)	3.58 ± 0.50	3.07 ± 0.39	0.01

MAA (cm)	3.91 ± 0.62	3.13 ± 0.45	0

Aortic stenoss	None: 11; Mild: 1	-	-

Aortic insufficiency	None: 9; Trace: 2; Mild: 1	-	-

## Conclusions

A semi-automated protocol for the combined analysis of the aortic valve geometry and hemodynamics in the aorta was developed to analyze MRI data obtained with 2D cine and 4D flow MRI. Application of this protocol and a preliminary analysis performed on the subset cohort of 12 BAV and 12 control cases revealed elevated mean velocity, jet angle and forward flow displacement in BAV subjects in the proximal ascending aorta. This study provides a roadmap for comprehensive large cohort assessment of correlations between geometry and hemodynamics associated with BAV disease.

## Funding

NHLBI grant R01HL115828, K25HL119608 and PURA scholarship.

